# Defining Delayed Perihematomal Edema Expansion in Intracerebral Hemorrhage: Segmentation, Time Course, Risk Factors and Clinical Outcome

**DOI:** 10.3389/fimmu.2022.911207

**Published:** 2022-05-09

**Authors:** Yihao Chen, Chenchen Qin, Jianbo Chang, Yixun Liu, Qinghua Zhang, Zeju Ye, Zhaojian Li, Fengxuan Tian, Wenbin Ma, Junji Wei, Ming Feng, Shengpan Chen, Jianhua Yao, Renzhi Wang

**Affiliations:** ^1^Department of Neurosurgery, Peking Union Medical College Hospital, Peking Union Medical College, Chinese Academy of Medical Sciences, Beijing, China; ^2^Tencent AI Lab, Shenzhen, China; ^3^Department of Neurosurgery, Shenzhen Nanshan Hospital, Shenzhen, China; ^4^Department of Neurosurgery, Dongguan People’s Hospital, Dongguan, China; ^5^Department of Neurosurgery, The Affiliated Hospital of Qingdao University, Qingdao, China; ^6^Department of Medicine, Qingdao University, Qingdao, China; ^7^Department of Neurosurgery, Qinghai Provincial People’s Hospital, Xining, China; ^8^Department of Neurosurgery, Guangdong Provincial People’s Hospital, Guangdong Institute of Neuroscience, Guangdong Academy of Medical Sciences, Guangzhou, China

**Keywords:** intracerebral hemorrhage, delayed perihematomal edema expansion, time course, prognosis, deep learning

## Abstract

We attempt to generate a definition of delayed perihematomal edema expansion (DPE) and analyze its time course, risk factors, and clinical outcomes. A multi-cohort data was derived from the Chinese Intracranial Hemorrhage Image Database (CICHID). A non-contrast computed tomography (NCCT) -based deep learning model was constructed for fully automated segmentation hematoma and perihematomal edema (PHE). Time course of hematoma and PHE evolution correlated to initial hematoma volume was volumetrically assessed. Predictive values for DPE were calculated through receiver operating characteristic curve analysis and were tested in an independent cohort. Logistic regression analysis was utilized to identify risk factors for DPE formation and poor outcomes. The test cohort’s Dice scores of lesion segmentation were 0.877 and 0.642 for hematoma and PHE, respectively. Overall, 1201 patients were enrolled for time-course analysis of ICH evolution. A total of 312 patients were further selected for DPE analysis. Time course analysis showed the growth peak of PHE approximately concentrates in 14 days after onset. The best cutoff for DPE to predict poor outcome was 3.34 mL of absolute PHE expansion from 4-7 days to 8-14 days (AUC=0.784, sensitivity=72.2%, specificity=81.2%), and 3.78 mL of absolute PHE expansion from 8-14 days to 15-21 days (AUC=0.682, sensitivity=59.3%, specificity=92.1%) in the derivation sample. Patients with DPE was associated with worse outcome (OR: 12.340, 95%CI: 6.378-23.873, P<0.01), and the larger initial hematoma volume (OR: 1.021, 95%CI: 1.000-1.043, P=0.049) was the significant risk factor for DPE formation. This study constructed a well-performance deep learning model for automatic segmentations of hematoma and PHE. A new definition of DPE was generated and is confirmed to be related to poor outcomes in ICH. Patients with larger initial hematoma volume have a higher risk of developing DPE formation.

## Introduction

Stroke is the second leading disease of death worldwide, annually causing 5.5 million deaths which half of the cases resulted from hemorrhagic stroke (1). The overall prognosis of patients with intracerebral hemorrhage (ICH) has not been significantly improved: the long-term mortality rate is 60% higher in ICH patients than patients without ICH (2) and with up to 80% of patients requiring long-term care (3, 4). Recently, basic and clinical-imaging research has confirmed that perihematomal edema (PHE) is a quantifiable marker of ICH’s secondary brain injury (SBI), associated with short-term or long-term prognosis (5–7). The PHE is considered a potential therapeutic target.

**Figure 1:**
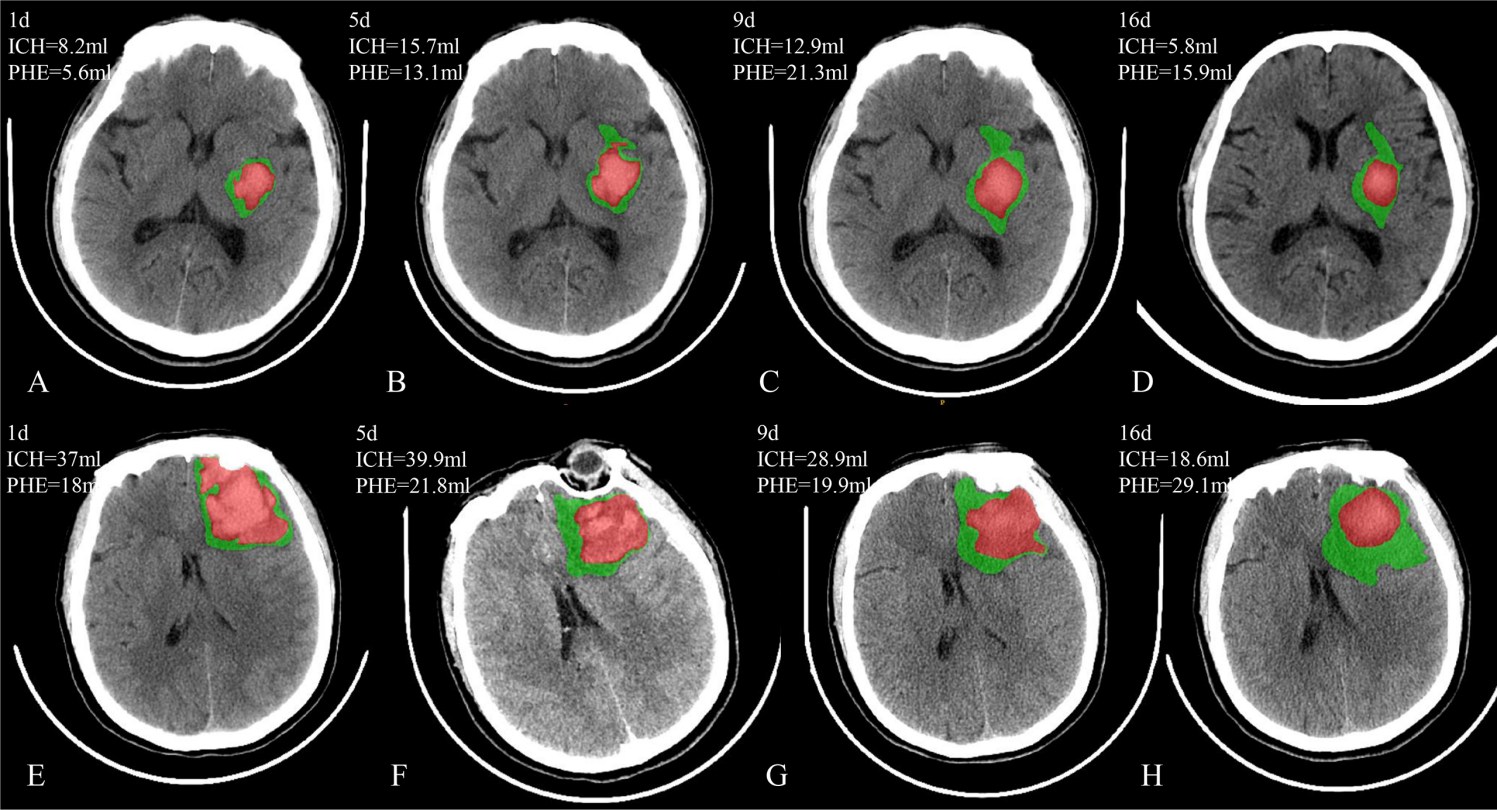
Two patients with PHE expansion in a delayed course. Case 1 **(A–D)** has the delayed PHE expansion from day 5 to day 9 after onset; Case 2 **(E–H)** has the delayed PHE expansion from day 9 to day 16 after onset.

The PHE develops rapidly in the early phase of ICH, resulting from the dysfunction of ion pumps in endothelial cells (8, 9), inflammatory reactions (10, 11), and hemoglobin cytotoxicity (12). Time within 72 hours of stroke onset has been considered the window for the rapid growth of PHE (13–15), and numerous studies have confirmed that the early PHE expansion is associated with a poor prognosis of ICH (5, 16, 17). The PHE grows with the decreased rate after the rapid phase and peaked at 1-2 weeks after onset (18, 19). However, a phenomenon of PHE expanding and peaking at a delayed course has also been reported (13, 20)(**Figure 1**). In clinical practice, delayed PHE expansion is usually required to be identified for a patient who represents the unexpected appearance of intracranial hypertension in the recovery phase of ICH, and therapy targeted for PHE induced intracranial hypertension is needed. However, studies exclusively focusing on delayed perihematomal edema expansion (DPE) are to date very sparse as research on long course PHE is present with limitations and challenges: 1) there have not yet been any methods measuring the volume of PHE [manual/semiautomatic (21, 22)/automatic approach (23, 24)] being considered to be convenient and reliable, which limits obtaining the data of PHE volume from a large sample size; 2) growth course for PHE is long and variable among individuals (25); 3) currently no consensus exists regarding the definition of DPE. Peng et al. (26) showed that patients who had a PHE volume in 12-20 days that was 3 mL greater than that within 5-9 days after ICH are at higher risk for poor prognosis. However, this is a predefinition for DPE, which needs validation. Furthermore, the sample size from this study is limited. 4) The relationship between DPE and prognosis is still unknown, and the amount of DPE necessary to produce poor outcomes is unclear.

To address the aforementioned unresolved issues, we constructed a non-contrast computed tomography (NCCT) based deep learning model for automated segmentation of hematoma and PHE. We further manually verified and refined all segmentations. The time course curve for ICH was plotted according to different initial hematoma volumes. We generated a new definition of DPE based on growth characteristics of edema and the best cut-off points of DPE volume associated with prognosis. We validated the relationship between DPE and poor outcomes and screened the DPE risk factors.

## Materials and Methods

### Study Population

A retrospective multicenter cohort of patients with spontaneous intracerebral hemorrhage (sICH) obtained from the Chinese Intracranial Hemorrhage Image Database (CICHID) was analyzed. The CICHID program began in 2019, led by Peking Union Medical College Hospital and supported by the Group of Medical Data, Chinese Medical Doctor Association. Seven participating centers were included in our cohort, and patients with available sICH data between 2016 and 2020 were identified as candidates for the current analyses. Strict inclusion and exclusion criteria were applied (see below). Any patients with missing data (essential clinical data such as age, sex, prognostic information) were retrieved and recorded.

### Inclusion and Exclusion Criteria

#### Inclusion Criteria

1) type of hemorrhage is limited with spontaneous intracerebral hemorrhage; 2) medical records are complete; 3) supratentorial hemorrhage; 4) patients have the initial NCCT scan within 24 hours after onset.

#### Exclusion Criteria

1) intraventricular hemorrhage (IVH) without cerebral parenchymal hemorrhage; 2) secondary causes of hemorrhage (e.g., trauma/brain tumor/cerebral infarction/aneurysm/vascular malformation/coagulation disorders); 3) hematoma removal approach; 4) low-quality imaging data (e.g., significant artefact).

In order to conduct DEP analysis, after generating the inclusion and exclusion criteria mentioned above, we further select patients have follow-up NCCT meet any of the following criteria: 1) have follow-up NCCT within 4-7d and 8-14d; 2) have follow-up NCCT within 8-14d and 15-21d.

### The Deep Learning Model for Image Segmentation

Overall, 859 NCCT scans with manual ICH segmentations were used to develop the deep learning model for automatic segmentation. We randomly separated the dataset into a training set, validation set, and test set with a ratio of 3:1:1. The validation set was used to determine the best hyper-parameter set for the training set, and the test set was used to evaluate the performance.

**Figure 2:**
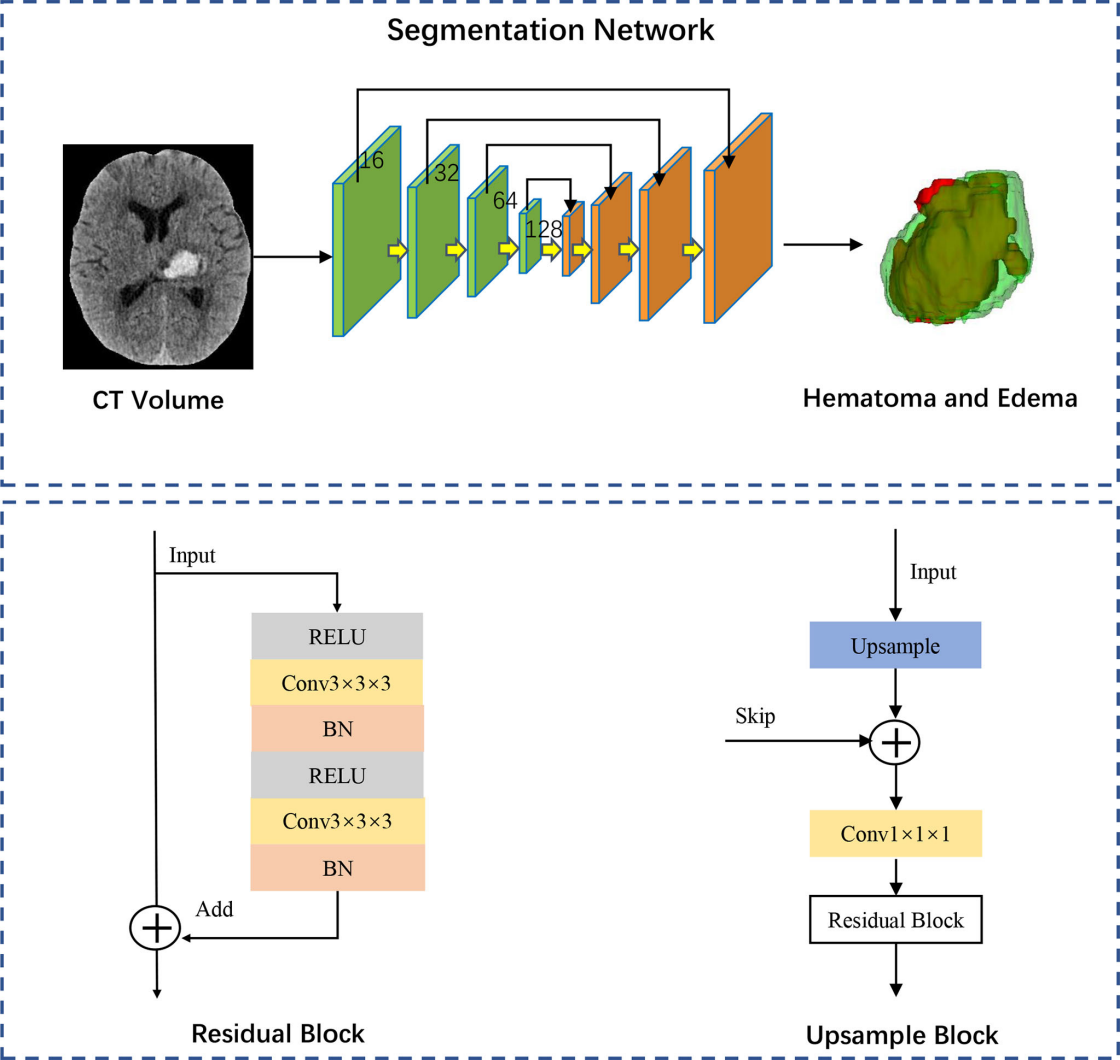
Network architecture of segmentation model. The 3D-Unet structure comprises a down-sampling path and an up-sampling path and each path in the network comprised the same four modules. The green down-sampling path comprises the Residual Block and a 1x1 convolution with versus 2. The feature maps with channels of 16, 32, 64 and 128 are gradually generated through the down-sampling path. The orange up-sampling path is comprised of the skip connections and Upsample Block. Each same scale of feature maps is skip connected during the up-sampling procedure. Feature maps with strengthened semantic information are generated with Element-wise. The segmentation network finally outputs the features with three channels activated by the Softmax layer and transformed into a probability map of background, hematoma, and edema. RELU, rectified linear unit; BN, batch normalization.

We constructed a fully convolutional network-3D Unet, demonstrated as a robust and efficient algorithm for medical image segmentation. The structure of the network is shown in **Figure 2**. The segmentation network finally outputs the features with three channels activated by the Softmax layer and transformed into a probability map of background, hematoma, and edema. Given that the PHE grows around hematoma, we constructed models for segmentation of hematoma and PHE separately, and we further regarded the combination lesion of hematoma and PHE as a completed region of interest for training. Detailed methods of the model’s construction were described in **Supplementary Methods**.

### Post-Processing of Automatic Segmentation and Demographics Data

**Figure 3:**
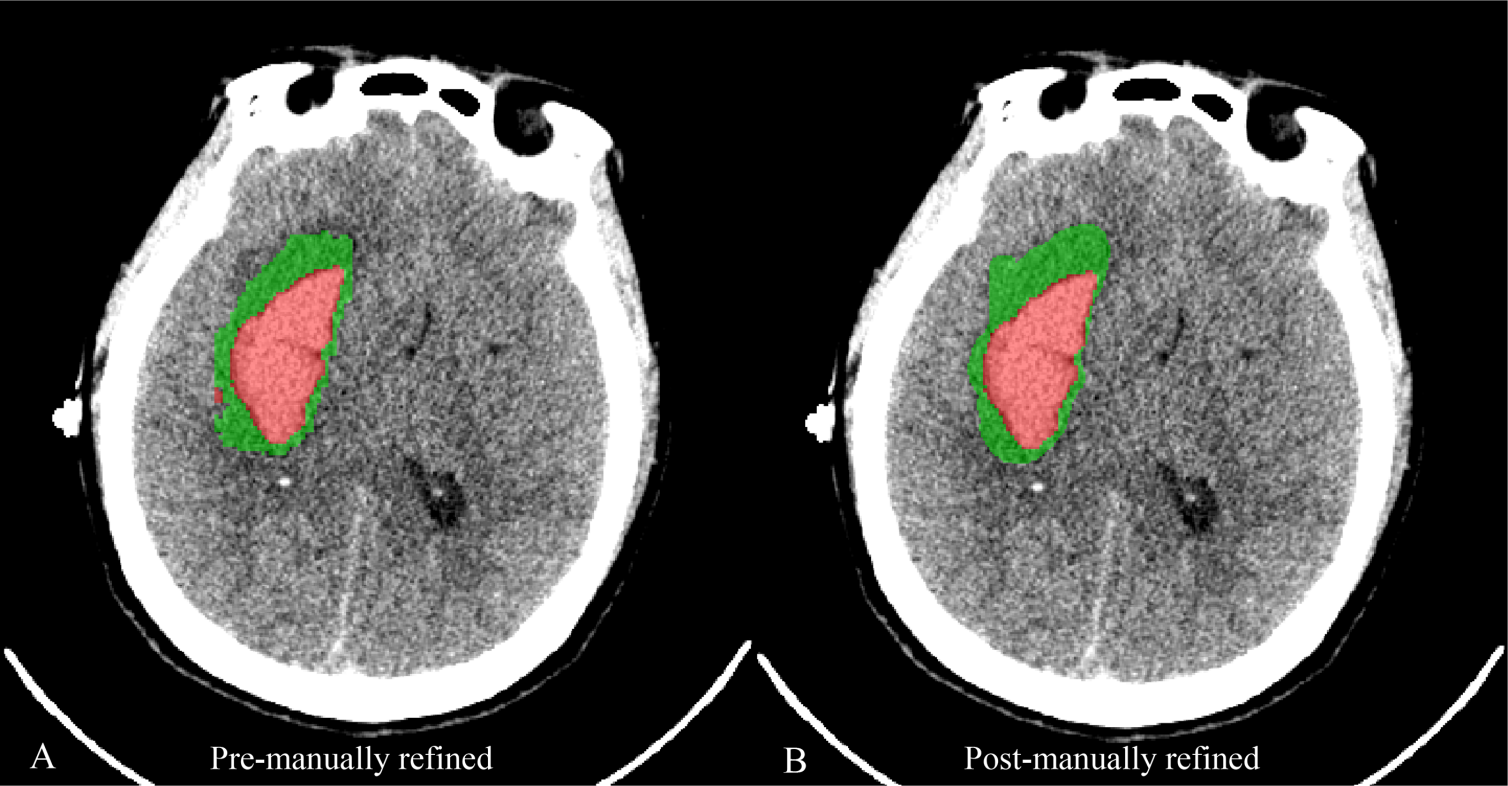
**A** case reveals the image of automatic segmentation for hematoma (red) and PHE (green) **(A)** and the corresponding image of segmentation being manually refined **(B)**.

The imaging and demographic data were fully anonymized prior to the analyses. All the automatic segmentations of hematoma and PHE were manually checked and refined by two experienced researchers [neurosurgeon (Y.C., 4 years of experience; J.C., 5 years of experience)] using “Insight Toolkit SNAP (ITK-SNAP)” software (27) (**Figure 3**). Clinical and demographic data were collected using a prespecified case report form (CRF) and were recorded independently by two researchers [neurosurgeon (Y.C., 4 years of experience; J.C., 5 years of experience)]. The data were reviewed for completeness and accuracy by another trained researcher [neurosurgeon (Z.Y., 10 years of experience)], who also re-acquired and recorded any relevant missing data.

### Time Course

The volume clusters of initial hematoma were defined for time-course analysis of ICH evolution: <10mL, 10-30mL, >30mL. Given that time points of NCCT acquisition are not identical across participants, we categorized follow-up imaging time frames: Day 1, Day 2 to 3, Day 4 to 6, Day 7 to 9, Day 10 to 12, Day 13 to15, Day 16 to 18, and Day 19 to 21.

### Outcome

The outcome assessed by using the modified Rankin Scale (mRS) was determined at discharge. A certified stroke specialist [neurosurgeon (J.W., 20 years of experience)] assessed and recorded the mRS score. A mRS score of 3-6 at discharge was defined as a poor outcome (28).

### DPE Definition

To determine the specific time window for DPE definition, we generated the time course curve and identified PHE growth’s peak and stable phase. A receiver operating characteristic (ROC) curve was generated to evaluate the prognostic predictive performance to determine the absolute edema expansion volume for DPE definition. As in other studies for hematoma expansion (29), the definition of DPE were derived from the thresholds at which PHE expansion was visually presented on sequential CT scans in delayed course. The method of Youden (30) was used to select the optimal cutoff point for DPE formation in the derivation sample (approximately 2/3 of the cohort) we randomly selected, and these cutoff points were then tested in the remaining test sample. We calculated sensitivity, specificity, and area under curve (AUC) values. The ROC curve analysis was performed with GraphPad Prism software (version 9.0).

### Statistical Analyses

Data analyses were performed using SPSS 24.0 (IBM Corp., Armonk, NY, USA). Normally distributed data were presented as the mean (± standard deviation) and compared groups using a Student’s t-test or a one-way ANOVA. Non-normally distributed data were presented as the median (and interquartile range [IQR]) and compared groups using a Mann–Whitney U test or Kruskal-Wallis H test. Categorical data were presented as the number of cases, the rate, or the constituent ratio (%), and comparisons used the chi-square test. We performed univariate analysis to explore the association between DPE and outcomes and screen DPE risk factors. The potential confounders include demographics, comorbidities, physical examinations, and imaging features. Factors that significantly predicted poor outcomes and DPE formation in univariate analyses were then included in a multivariate logistics regression analysis. A two-tailed P < 0.05 was considered statistically significant.

## Results

### Performance of Lesion Segmentation

For segmentation of hematoma and PHE trained separately, the validation cohort achieved Dice scores of 0.903 ± 0.086 and 0.665 ± 0.136 for hematoma and PHE, respectively. The corresponding values for the test cohort were 0.880 ± 0.135 and 0.639 ± 0.148, respectively. For segmentation of the hematoma and PHE being regarded as a completed region of interest that is trained simultaneously, the validation cohort achieved Dice scores of 0.901 ± 0.088 and 0.670 ± 0.129 for hematoma and PHE, respectively. The corresponding values for the test cohort were 0.877 ± 0.129 and 0.642 ± 0.148, respectively. The results of lesion segmentation performance are shown in **Table 1**.

**Table 1:** Lesion Segmentation Performance on Validation and Test Datasets.

Cohort	Hematoma Dice	Hematoma CC	Hematoma CCC	PHE Dice	PHE CC	PHE CCC
Validation						
H&P separate	0.903 (0.086)	0.994 (0.010)	0.985 (0.080)	0.665 (0.136)	0.945 (0.117)	0.890 (0.143)
H&P integrate	0.901 (0.088)	0.993 (0.011)	0.983 (0.080)	0.670 (0.129)	0.953 (0.075)	0.890 (0.133)
Test						
H&P separate	0.880 (0.135)	0.976 (0.105)	0.965 (0.140)	0.639 (0.148)	0.943 (0.094)	0.870 (0.169)
H&P integrate	0.877 (0.129)	0.977 (0.096)	0.965 (0.129)	0.642 (0.148)	0.949 (0.067)	0.865 (0.176)

“H&P separate” indicates models for segmentation of hematoma and PHE trained separately;

“H&P integrate” indicates model for segmentation of the combination lesion, which the hematoma and PHE being regarded as a completed region of interest that is trained simultaneously; CC, the Pearson product-moment correlation coefficient; CCC, concordance correlation coefficient.

### Study Population and Time Course

**Figure 4:**
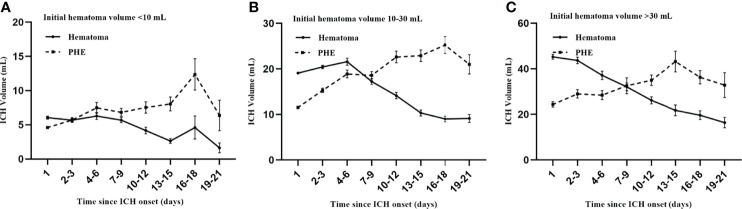
Time course of ICH evolution. The growth pattern for PHE **(A–C)** is more variable compared to the hematoma **(A–C)**. Patients have a significant final peak PHE formed after approximately two weeks **(A–C)**. The PHE grows slowly after the first week in patients with an initial hematoma volume of less than 30 mL **(A, B)**. Similarly, the PHE maintains a stable growth rate after the first week in patients with initial hematoma larger than 30 mL **(C)**.

Overall, 1201 patients with spontaneous ICH from 7 participating centers were enrolled for time-course analysis of ICH evolution. **Figure 4** illustrates the time-course curve for different initial hematoma volumes. The growth pattern for hematoma is characterized as stable or decreased over time, whether patients have a small or large initial hematoma (**Figure 4A-C**). Compared to the hematoma, the PHE grows variable in a long course. A final peak PHE was approximately formed in the time of 14 days after onset (**Figure 4A-C**). The PHE grows slowly after the first week for patients with an initial hematoma volume of less than 30 mL (**Figure 4A, B**). Similarly, the PHE maintained a stable growth rate after the first week in patients with initial hematoma larger than 30 mL (**Figure 4C**).

From the growth curve, we identified two windows for stable phase of PHE: 8-14 days and 15-21days after onset, which can be used to determine the time window for DPE. Consequently, for DPE analysis, we further selected 312 patients who had follow-up NCCT meeting any of the following criteria:1) had follow-up NCCT within 4-7d and 8-14d; 2) had follow-up NCCT within 8-14d and 15-21d. Patients who had abnormal PHE expansion across any of these continuous windows were eligible to be defined as DPE formation. The DPE population (n = 312) was divided into a derivation sample (n = 214) and a test sample (n = 98).

### Prognostic Predictive Value of DPE

**Figure 5:**
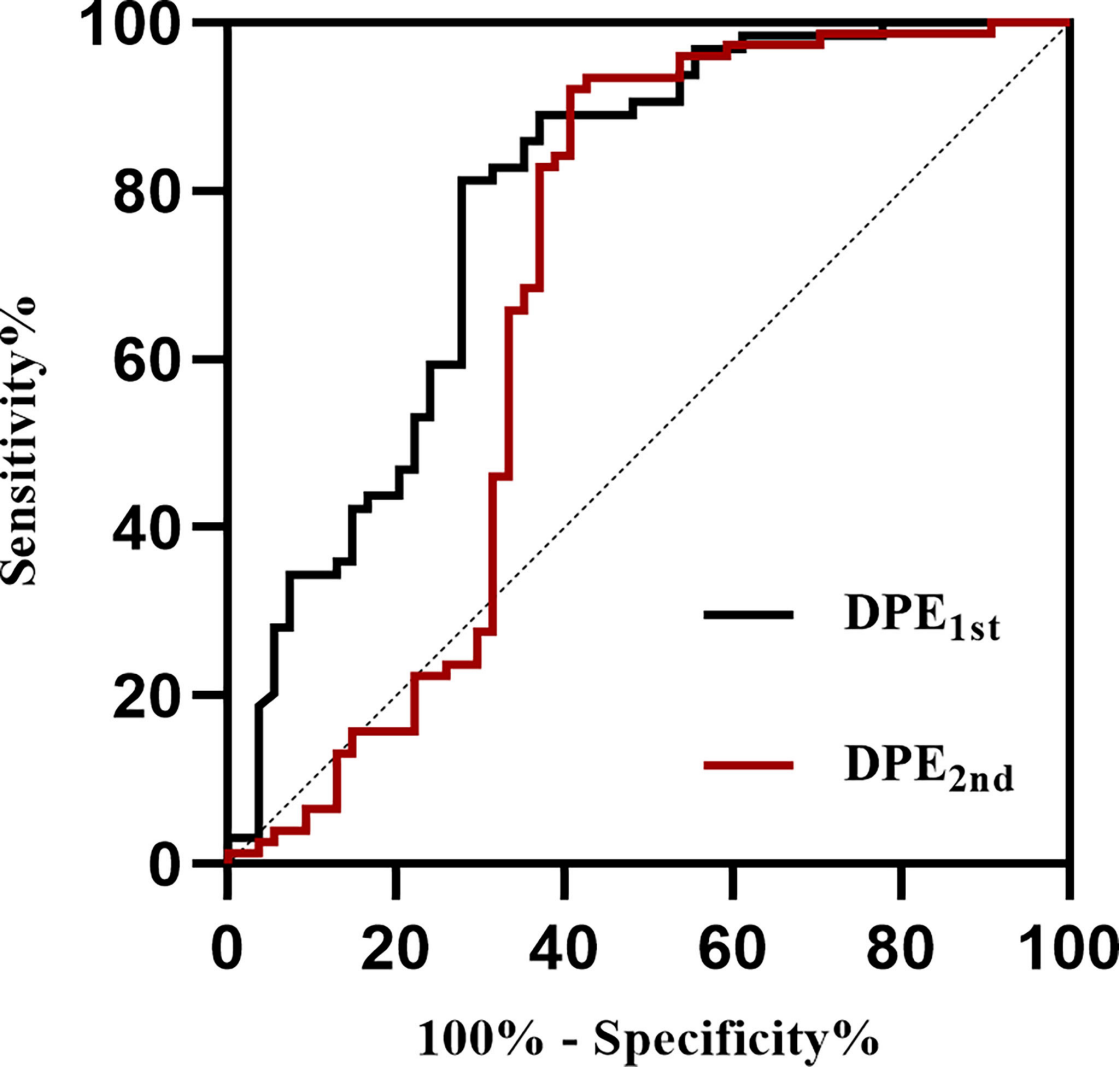
Receiver operating characteristic curves of DPE to identify patients with poor outcomes at discharge. DPE, delayed perihematomal edema expansion. DPE_1st_ indicates DPE from 4-7days to 8-14days, and DPE_2nd_ indicates DPE from 8-14days to 15-21days.

ROC curves for DPE from 4-7days to 8-14days (DPE_1st_) and DPE from 8-14days to 15-21days (DPE_2nd_) for the prediction of poor outcome in derivation sample are shown in **Figure 5**. According to the method of Youden, the best cutoff point of DPE_1st_ volume was 3.34 mL (AUC=0.784, 95%CI 0.698-0.854, **Table 2**) with a sensitivity of 72.2% and a specificity of 81.2%; and the best cut-off point of DPE_2nd_ volume was 3.78 mL (AUC=0.682, 95%CI 0.594-0.760, **Table 2**) with a sensitivity of 59.3% and a specificity of 92.1%. The two cutoff points performed well in the test sample: the DPE_1st_ (>3.34 mL) has a sensitivity of 67.4% and specificity of 90.9% with an AUC of 0.792. The DPE_2nd_ (>3.78 mL) has a sensitivity of 32.2% and specificity of 100%, with an AUC of 0.661. For patients with DPE_1st_ (>3.34 mL) or DPE_2nd_ (>3.78 mL), the predictive value of sensitivity is 54.5%, specificity is 93.7%, and the AUC is 0.741 (**Table 2**).

**Table 2:** Predictive Values of DPE for The Identification of Patients with Poor Prognosis in The Derivation Sample and The Test Sample.

Variable	P value	Sensitivity%	Specificity%	AUC	95%CI	best cutoff point (mL)
Derivation sample						
DPE_1st_	<0.01	72.2	81.2	0.784	0.698-0.854	3.34
DPE_2nd_	<0.01	59.3	92.1	0.682	0.594-0.760	3.78
Test sample						
DPE_1st_ (>3.34 mL)	<0.01	67.4	90.9	0.792	0.673-0.883	–
DPE_2nd_ (>3.78 mL)	<0.01	32.2	100	0.661	0.510-0.792	–
DPE_1st_ (>3.34 mL) or DPE_2nd_ (>3.78 mL)	<0.01	54.5	93.7	0.741	0.643-0.825	–

DPE, Delayed perihematomal edema expansion; AUC, Area under the curve; CI, Confidence interval.

### Baseline Characteristics and Risk Factors of DPE

In the cohort including 312 patients for DPE analysis, there were 211 males (67.62%) and 101 females (32.38%), with a median age of 57 (IQR 49-67) years old. The median volume of initial hematoma was 20.82 (IQR 12.59-31.78) mL, and the volume of initial PHE was 8.88 (IQR 5.63-14.43) mL. A total of 253 patients (81.09%) had deep ICH, and 59 patients (18.91%) had lobar ICH. Overall, 164 patients (52.56%) had a poor outcome, and a total of 124 patients (39.74%) had DPE_1st_ (>3.34 mL) or DPE_2nd_ (>3.78 mL) that was defined as DPE formation and enrolled into the DPE group in this study. Univariate analysis showed that the DPE and Non-DPE groups differed significantly in GCS on admission, initial hematoma volume, and the initial PHE volume (all P<0.05, see **Table 3**). Multivariate logistic regression analysis finally identified that only the larger initial hematoma volume (OR: 1.021, 95%CI: 1.000-1.043, P=0.049) was the significant risk factor for DPE formation, but not the GCS on admission (OR: 0.925, 95%CI: 0.843-1.016, P=0.102) and not the initial PHE volume (OR: 1.019, 95%CI: 0.983-1.057, P=0.310).

**Table 3:** Baseline Demographic and Clinical Characteristics of Patients in DPE Group and The Non-DPE Group.

Variables	DPE group	Non-DPE group	P value
	n=124 (39.74%)	n=188 (60.26%)	
Age, years	58 (51-67)	56 (48-67)	0.230
Male sex, n (%)	84 (67.74%)	127 (67.55%)	0.972
GCS on admission	13 (11-14)	14 (12-15)	<0.01*
Comorbidities, n (%)			
History of hemorrhagic stroke	13 (10.48%)	10 (5.32%)	0.088
History of ischemic stroke	15 (12.09%)	16 (8.51%)	0.300
Hypertension	95 (76.61%)	138 (73.40%)	0.524
Diabetes mellitus	13 (10.48%)	19 (10.10%)	0.914
Coronary heart disease	8 (6.45%)	9 (4.78%)	0.526
Anticoagulant therapy	2 (1.61%)	4 (2.12%)	1.000
Anti-platelet therapy	7 (5.64%)	11 (5.85%)	0.939
Smoking	30 (24.19%)	42 (22.34%)	0.704
Alcohol intake	26 (20.96%)	34 (18.08%)	0.527
Physical examination, mmHg			
Systolic blood pressure	169 (150-185)	169 (151-186)	0.792
Diastolic blood pressure	98 (86-110)	100 (87-110)	0.697
CT image			
Initial hematoma volume, ml	23.24 (15.49-35.11)	18.72 (11.94-27.60)	<0.01*
Initial PHE volume, ml	10.09 (6.35-16.88)	8.02 (4.58-13.56)	<0.01*
ICH locations			0.469
Deep ICH	103 (83.06%)	150 (79.78%)	–
Lobar ICH	21 (16.94%)	38 (20.22%)	–
Poor outcome	104 (83.87%)	60 (31.91%)	<0.01*

DPE, Delayed perihematomal edema expansion; GCS, Glasgow coma scale; PHE, Perihematomal edema; ICH, Intracerebral hemorrhage.

*Indicates P value <0.05.

Patients with DPE_1st_ (>3.34 mL) or DPE_2nd_ (>3.78 mL) were included in the DPE group.

### Relationship Between DPE and Outcome

We generated another multivariate logistic regression analysis to confirm whether the DPE formation [DPE_1st_ (>3.34 mL) or DPE_2nd_ (>3.78 mL)] independently affects the prognosis in sICH patients. Univariate analyses showed that the group of a favorable outcome and group of poor outcome differed significantly in age, GCS score on admission, history of hemorrhagic stroke/ischemic stroke/coronary heart disease/anti-platelet therapy, initial hematoma/PHE volume, deep ICH, and DPE formation (all P < 0.05, see **Supplementary Table 1**).

Multivariate regression analyses showed that a poor prognosis was associated with advanced age (OR: 1.049, 95% CI: 1.022-1.077, P < 0.01), a lower GCS score on admission (OR: 0.746, 95% CI: 0.646-0.861, P < 0.01), a larger initial hematoma volume (OR: 1.043, 95% CI: 1.009-1.079, P = 0.014), deep ICH (OR: 2.907, 95% CI: 1.188-7.112, P = 0.019), and DPE formation (OR: 12.340, 95% CI: 6.378-23.873, P < 0.01), as shown in **Table 4**.

**Table 4:** Multivariable Logistic Regression Analysis of Patients with Poor Prognosis.

Variable	P value	OR	OR 95% CI
Age	<0.01*	1.049	1.022-1.077
GCS on admission	<0.01*	0.746	0.646-0.861
History of hemorrhagic stroke	0.065	3.547	0.926-13.587
History of ischemic stroke	0.760	1.205	0.364-3.987
Coronary heart disease	0.133	3.178	0.703-14.368
Anti-platelet therapy	0.244	2.462	0.541-11.195
Initial hematoma volume	0.014*	1.043	1.009-1.079
Initial PHE volume	0.685	1.011	0.958-1.068
Deep ICH	0.019*	2.907	1.188-7.112
DPE formation	<0.01*	12.340	6.378-23.873

GCS, Glasgow coma scale; PHE, Perihematomal edema; ICH, Intracerebral hemorrhage; DPE, Delayed perihematomal edema expansion;

*Indicates P value <0.05.

DPE formation indicates DPE_1st_ (>3.34 mL) or DPE_2nd_ (>3.78 mL).

## Discussion

This study innovatively devised and framed a deep learning model for automatic segmentation of hematoma and PHE. We identified the growth peak of 14 days and two stable phases of 8-14 days and 15-21 days by plotting a corresponding time-course curve versus different initial hematoma volumes. We determined that volume of absolute PHE in 8-14 days is 3.34 mL larger than that in 4-7 days, or the volume of absolute PHE in 15-21 days is 3.78 mL larger than that in 8-14 days can be well defined as DPE formation as it is associated with poor outcome. We identified that the initial hematoma volume independently affects DPE formation.

The PHE expansion has been considered a novel neuroimaging marker of poor prognosis, and a recent study reviewed the impact of PHE on ICH prognosis (25). The pre-clinical research on magnetic resonance spectroscopy revealed that the recovery of N-acetylaspartate, choline, and creatine was faster in the PHE area than in the non-PHE area (31), suggesting that the PHE may provide a protective buffer against irreversible impairment. Nevertheless, real-world research showed that patients with absolute (15, 32, 33) and relative PHE expansion (5, 16) have a higher risk of developing poor neurological outcomes. Compared to the well-established research for hematoma expansion, we believe the PHE expansion is worthy of further investigation based on its good sensitivity for prognosis prediction, and a more comprehensive prediction model for PHE expansion is needed.

Any PHE study presents challenges for measuring the volume of edema. Manual segmentation for PHE is reliable but laborious. Method of CT value-based semiautomatic segmentation (21) and the deep learning-based automatic segmentation (23, 24) have a promising consistency performance but are characterized by poor accuracy. To address the issues, we constructed a deep learning model for automated segmentation of hematoma and edema simultaneously, and the segmentations were further manually refined. The innovation of this work lies in that we regarded the combination lesion of hematoma and PHE as a completed region of interest for training, which effectively limits the possibility of segmentation error like the separation of hematoma and PHE, and improves the segmentation accuracy. To the best of our knowledge, we are the first to report a deep learning-based model of simultaneous segmentation for the total lesion of ICH.

Many studies confirmed that PHE develops rapidly within 3 days after stroke onset (13–15). However, controversy exists on the peak phase of PHE growth, which could be the time of 1 week (18, 19), 2 weeks (13, 34), and even 3 weeks (20). Beyond the individual differences accounting for PHE growth, the reason for previous research reporting different peaks of PHE may result from patients with mixed baseline hematoma being enrolled for time-course analysis, and the baseline hematoma is associated with PHE promotion (14, 17, 35). Resultantly, we plotted the time course curve for ICH according to different initial hematoma volumes. The time curve shows the final peak concentrated on approximately 2 weeks after onset, no matter how large the baseline hematoma was. This finding agrees with the previous experiment reported by Sprügel et al. (7). It’s unexpected to note that a small peak firstly formed before the stable phase of 8-14 days after onset in patients with baseline hematoma smaller than 30 mL. We considered this finding significant as the time-course curve displayed the variability of PHE growth, which opposed the established theory that PHE remains steady after a specific peak. This finding reminds clinicians to pay attention to the abnormal PHE expansion in the delayed phase of ICH recovery in clinical practice.

There is no consensus definition for DPE currently. Peng et al. (26) pre-defined DPE as PHE volume in 12 to 20 days is 3 mL larger than that in 5 to 9 days, and identify patients who meet the criteria have a higher risk of poor outcome. However, a further comprehensive evaluation for predictive performance and a relevant external test is lacking; and the significant variable timing of follow-up CT (between day 5-day 20) might affect the predictive performance (sensitivity and specificity) of such definition of DPE. In the present study, we detailed the time window for DPE based on the growth curve of the PHE cohort. Given the course for PHE growth is long, we determined two optimal cutoff points of DPE volume across two different time windows. Compared to the derivation sample, the two cutoff points reproduced well in the test sample except for somewhat lower sensitivity for the DPE_1st_ and DPE_2nd_. We speculate that the number of targeted cases that meet the cutoff points in the test sample is small, which may be relevant for the predictive performance of cutoff points in the test sample. We recommend future prospective studies enroll a larger sample size to explain this phenomenon. Given that the neurological deficits may partially improve over time, we agree that a long-term prognosis makes more sense than a short-term prognosis. However, because up to 80% of ICH patients cannot maintain an independent living after six months (3, 4), we opined that the mRS at discharge well represents trends of rehabilitation among patients and could be reasonably utilized for DPE definition.

Few clinical or imaging markers have been identified to predict DPE. Venkatasubramanian et al. (13) confirmed the patient with a high baseline hematocrit is associated with delayed peak PHE. This may be because a high hematocrit indicates higher red blood cell degradation, which has been identified as an important mechanism for PHE promotion (36). Peng et al. (26) demonstrated a large baseline hematoma and age were significant risk factors for PHE expansion in delayed course. Our study also revealed that a large hematoma independently affects DPE, but advanced age is not. We opined the hypothesis that elderly patients with brain atrophy have lower odds of PHE promotion needs further validation as the mechanism is poorly understood. Sprügel et al. (7) reported that patients with lobar ICH have earlier PHE peak than deep ICH, which may be due to the looseness characteristics in different brain structures. However, this present study did not find the ICH location (supratentorial ICH) is associated with DPE formation. We recommend further using imaging radiomics with prior interpretability to identify more imaging markers and construct a robust imaging based deep learning model for DPE prediction.

The major limitations of this paper are the following. First, the MRI modality would contribute more to PHE segmentation and its volume calculation. However, the MRI is not routinely used in clinical management for ICH patients but could be a critical future direction. Second, time points of NCCT acquisition are not identical across participants in this retrospective study, which could have potentially affected the time course analysis for PHE growth. Thus, the next step should be the organization of prospective clinical trials with a detailed imaging acquisition protocol. Finally, further studies could analyze the predictive value of multiple biomarkers like inflammation indicators.

Overall, a well-performance deep learning model for segmentation of hematoma and PHE was constructed in this study. We identified the peak of 14 days after onset and two stable phases of 8-14 days and 15-21 days for PHE growth. DPE with the new definition generated in this study robustly predicts the poor outcome of ICH. Patients with larger initial hematoma volume have a higher risk of developing DPE.

## Data Availability Statement

The original contributions presented in the study are included in the article/**Supplementary Material**. Further inquiries can be directed to the corresponding authors.

## Ethics Statement 

This retrospective study was approved by the Institutional Review Board of Peking Union Medical College Hospital (PUMCH; Ethics code: S-K1175). Written informed consent for participation was not required for this study in accordance with the institutional requirements.

## Author Contributions

YC and RW conceived and designed the study. YC, CQ, and JC collected and analyzed data, and wrote the manuscript. CQ and YL constructed the deep learning model for automatic ICH segmentation. All the authors collected and analyzed data. WM, JW, MF, SC, JY, and RW revised the manuscript. All authors contributed to the article and approved the submitted version.

## Funding

This work was supported by the National Key R&D Program of China (2018YFA0108603), the Beijing Tianjin Hebei basic research cooperation project (19JCZDJC64600(Z)), the CAMS Innovation Fund for medical sciences (CIFMS) (2020-I2M-C&T-B-028), the National Natural Science Foundation of China (82001389).

## Conflict of Interest

Authors CQ and YL were employed by Tencent.

The remaining authors declare that the research was conducted in the absence of any commercial or financial relationships that could be construed as a potential conflict of interest.

## Publisher’s Note

All claims expressed in this article are solely those of the authors and do not necessarily represent those of their affiliated organizations, or those of the publisher, the editors and the reviewers. Any product that may be evaluated in this article, or claim that may be made by its manufacturer, is not guaranteed or endorsed by the publisher.

## Abbreviations

PHE, perihematomal edema; SBI, secondary brain injury; ICH, intracerebral hemorrhage; DPE, delayed perihematomal edema expansion; CICHID, Chinese Intracranial Hemorrhage Image Database; NCCT, non-contrast computed tomography; mRS, modified Rankin Scale; sICH, spontaneous intracerebral hemorrhage; CRF, case report form; ROC, receiver operating characteristic; AUC, area under curve.
