# Supplementary material for: Defining Delayed Perihematomal Edema Expansion in Intracerebral Hemorrhage: Segmentation, Time Course, Risk Factors and Clinical Outcome
Source: Front Immunol. 2022 May 9;13:911207. doi: 10.3389/fimmu.2022.911207 (PMC9125313; doi:10.3389/fimmu.2022.911207)
Supplement: Supplementary file 1 [file DataSheet_1.docx]

SUPPLEMENTAL MATERIAL

**Methods of Automatic Lesion Segmentation**

1. **Dataset**

Overall, 859 NCCT scans with manual ICH segmentations were used to develop the deep learning model for automatic segmentation. We randomly separated the dataset into a training set, validation set, and test set with a ratio of 3:1:1. There are 176 NCCTs enrolled in the validation set, which was used to determine the best hyper-parameter set for the training set, and the test set of 176 NCCTs is used to evaluate the performance.

1. **Image processing**

The CT image inputs were skull stripped before training. Our own developed 3D Unet-based segmentation network was used for skull stripped. Invalid areas of the images are cropped off based on the Bounding Box of the brain. Due to the orientation of the CT image being variable when captured, all images were corrected to RAI orientation by ITK's reorient module and sampled to 176x176x176 size. The edema was characterized with minimal change of grayscale. Consequently, the network input brain window [0,80], edema window [5,35], and subdural window [-20,180] for easier extraction of edema features. The window processing equation is as follows:

$I_{out} = \frac{clip(I_{in}, w_{min}, w_{max}) - w_{min}}{w_{max} - w_{min}}$.

Of the function, the $I_{out}$ indicates output image, $w_{min}$ and $w_{max}$ indicates upper and lower bounds of the window. The clip function indicates that all CT values less than $w_{min}$ are set to $w_{min}$, and all CT values greater than $w_{max}$ are set to $w_{max}$. We obtained three images after normalization of CT windows: image of $I_{brain}$, $I_{edema}$ and $I_{subdural}$. To refined the generalization ability of the network, we adopt an online data augmentation strategy during training, like random flipping.

**3. Experiments Setup**

An Intel(R) Xeon(R) Platinum 8255C CPU with 24 Cores and an NVIDIA Tesla V100 GPU was used for training. We used Pytorch1.6.0 for our experiments. For optimization, we used Adam optimizer. The weight decay was set at $1\times{10}^{-5}$, and the learning rate was set at $3\times{10}^{-4}$. The network was trained up to 300 epochs. The model with the highest Dice of edema in the validation cohort was used for final training.

**4.** **Network Architecture**

The 3D-Unet structure comprises a down-sampling path and an up-sampling path and each path in the network comprised the same four modules. The green down-sampling path comprises the Residual Block and a 1x1 convolution with versus 2. The feature maps with channels of 16, 32, 64 and 128 are gradually generated through the down-sampling path. The orange up-sampling path is comprised of the skip connections and Upsample Block. Each same scale of feature maps is skip connected during the up-sampling procedure. Feature maps with strengthened semantic information are generated with Element-wise. The segmentation network finally outputs the features with three channels activated by the Softmax layer and transformed into a probability map of background, hematoma, and edema.

**5. Loss Function**

Given the segmentation for edema is relatively difficult, we integrated two functions of Cross Entropy and Dice Loss. The Dice Loss function is as follow:

$$L_{dice}=1-\frac{2\left| X\bigcap Y \right|}{\left| X \right|+ \left| Y \right|}$$

Of this function, the $X$indicates network output probabilities map, and the $Y$indicates the corresponding golden standard. Cross Entropy is as follow:

$$l_{ce}= -\frac{1}{N}\sum_{i=i}^{N} \sum_{c=i}^{C} w_{c}g_{i}^{c}log(p_{i}^{c})$$

$N$ is the number of pixels, $C$ is the number of categories. There are 3 categories, namely background, hematoma, and edema in our experiment. $g$ is the gold standard image and $p$ is the predicted image. There were three region weights which are 0.5, 1.0, and 1.0, respectively. The overall objective function for image segmentation is as follow:

$$L_{total}={\lambda_{1}L}_{ce} + {\lambda_{2}L}_{hematoma} + {\lambda_{3}L}_{edema}$$

Where $L_{total}$ is the total loss; $\lambda_{1}$ is the weight of Cross Entropy, which is 0.2. $\lambda_{2}$ is the weight of hematoma segmentation with a value of 0.25; $L_{hematoma}$ is the Dice Loss of hematoma, $\lambda_{3}$ is the segmentation weight of edema region with a value of 1.0, and $L_{edema}$ is the Dice Loss of edema. Cross Entropy is mainly used as a point constraint, which distinguished the foreground and background well. The Dice Loss is better to learn the target shape as a shape constraint. The combination of the two methods enabled the network to perform better in edema and hematoma segmentation tasks.
