# Supplementary material for: Defining Delayed Perihematomal Edema Expansion in Intracerebral Hemorrhage: Segmentation, Time Course, Risk Factors and Clinical Outcome
Source: Front Immunol. 2022 May 9;13:911207. doi: 10.3389/fimmu.2022.911207 (PMC9125313; doi:10.3389/fimmu.2022.911207)
Supplement: Supplementary file 2 [file Table_1.docx]

**Supplementary Table 1.** Univariate Analysis of Patients with Poor Prognosis

| **Variables** | **Favorable outcome**  **(mRS 0-2)** | **Poor outcome**  **(mRS 3-6)** | **P value** |
| --- | --- | --- | --- |
|  | **n=151(48.40%)** | **n=161(51.60%)** |  |
| Age, years | 54(46-65) | 60(52-69) | <0.01* |
| Male sex, n (%) | 100(66.22%) | 111(68.94%) | 0.983 |
| GCS on admission | 14(13-15) | 13(10-14) | <0.01* |
| Comorbidities, n (%) |  |  |  |
| History of hemorrhagic stroke | 4 (2.65%) | 19(11.80%) | <0.01* |
| History of ischemic stroke | 7(4.63%) | 24(14.90%) | <0.01* |
| Hypertension | 106(70.19%) | 127(78.88%) | 0.238 |
| Diabetes mellitus | 12(7.94%) | 20(12.42%) | 0.235 |
| Coronary heart disease | 3(1.98%) | 14(8.69%) | 0.011* |
| Anticoagulant therapy | 3(1.98%) | 3(1.86%) | 1.000 |
| Anti-platelet therapy | 4(2.65%) | 14(8.69%) | 0.027* |
| Smoking | 32(21.19%) | 40(24.84%) | 0.562 |
| Alcohol intake | 25(16.55%) | 35(21.74%) | 0.319 |
| Physical examination, mmHg |  |  |  |
| Systolic blood pressure | 166(150-185) | 170(150-187) | 0.718 |
| Diastolic blood pressure | 100(87-110) | 98(86-110) | 0.510 |
| CT image |  |  |  |
| Initial hematoma volume, ml | 15.85(10.82-25.53) | 24.02(15.85-34.72) | <0.01* |
| Initial PHE volume, ml | 7.77(4.91-13.46) | 10.08(6.14-16.09) | <0.01* |
| ICH locations |  |  | 0.020* |
| Deep ICH | 112(74.17%) | 141(87.57%) | - |
| Lobar ICH | 36(23.84%) | 23(14.28%) | - |
| DPE formation | 20(13.24%) | 104(64.59%) | <0.01* |

Abbreviations: mRS, modified Rankin Scale; GCS, Glasgow coma scale; PHE, Perihematomal edema; ICH, Intracerebral hemorrhage; DPE, Delayed perihematomal edema expansion

DPE formation indicates DPE_1st_ (>3.34 mL) or DPE_2nd_ (>3.78 mL)

*Indicates P value <0.05
